# Lincomycin HCl-Loaded Borneol-Based In Situ Gel for Periodontitis Treatment

**DOI:** 10.3390/gels9060495

**Published:** 2023-06-19

**Authors:** Napaphol Puyathorn, Nutdanai Lertsuphotvanit, Takron Chantadee, Wiwat Pichayakorn, Thawatchai Phaechamud

**Affiliations:** 1 Programme of Pharmaceutical Engineering, Department of Industrial Pharmacy, Faculty of Pharmacy, Silpakorn University, Nakhon Pathom 73000, Thailand; puyathorn_n@silpakorn.edu; 2 Program of Pharmaceutical Technology, Department of Industrial Pharmacy, Faculty of Pharmacy, Silpakorn University, Nakhon Pathom 73000, Thailand; lertsuphotvanit_n@silpakorn.edu; 3 Department of Pharmaceutical Sciences, Faculty of Pharmacy, Chiang Mai University, Chiang Mai 50200, Thailand; takron.chantadee@cmu.ac.th; 4 Natural Bioactive and Material for Health Promotion and Drug Delivery System Group (NBM), Faculty of Pharmacy, Silpakorn University, Nakhon Pathom 73000, Thailand; 5 Department of Pharmaceutical Technology, Faculty of Pharmaceutical Sciences, Prince of Songkla University, Songkhla 90110, Thailand; 6 Department of Industrial Pharmacy, Faculty of Pharmacy, Silpakorn University, Nakhon Pathom 73000, Thailand

**Keywords:** in situ forming gel, borneol, lincomycin hydrochloride, solvent exchange

## Abstract

Solvent exchange-induced in situ forming gel (ISG) has emerged as a versatile drug delivery system, particularly for periodontal pocket applications. In this study, we developed lincomycin HCl-loaded ISGs using a 40% borneol-based matrix and N-methyl pyrrolidone (NMP) as a solvent. The physicochemical properties and antimicrobial activities of the ISGs were evaluated. The prepared ISGs exhibited low viscosity and reduced surface tension, allowing for easy injection and spreadability. Gel formation increased the contact angle on agarose gel, while higher lincomycin HCl content decreased water tolerance and facilitated phase separation. The drug-loading influenced solvent exchange and matrix formation, resulting in thinner and inhomogeneous borneol matrices with slower gel formation and lower gel hardness. The lincomycin HCl-loaded borneol-based ISGs demonstrated sustained drug release above the minimum inhibitory concentration (MIC) for 8 days, following Fickian diffusion and fitting well with Higuchi’s equation. These formulations exhibited dose-dependent inhibition of *Staphylococcus aureus* ATCC 25923, *Escherichia coli* ATCC 8739, and *Prophyromonas gingivalis* ATCC 33277, and the release of NMP effectively inhibited *Candida albicans* ATCC 10231. Overall, the 7.5% lincomycin HCl-loaded 40% borneol-based ISGs hold promise as localized drug delivery systems for periodontitis treatment.

## 1. Introduction

**Figure 1:**
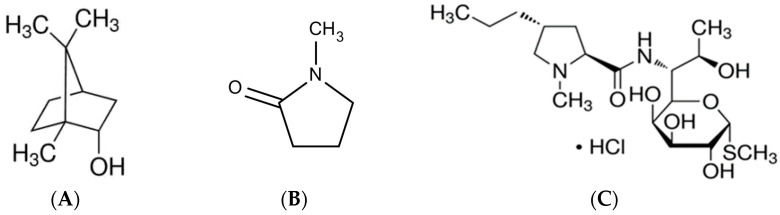
Chemical structures of borneol (**A**), NMP (**B**), and lincomycin HCl (**C**).

The matrix-forming agent in a solvent exchange-induced in situ forming gel (ISG) plays a crucial role and should possess properties such as insolubility in water, biocompatibility, biodegradability, and safety. Borneol, a lipid-soluble bicyclic monoterpene (Figure 1A), meets these criteria, as it is insoluble in water and has a long history of safe use in Traditional Chinese Medicine (TCM) [1,2]. Borneol has been recognized for its ability to enhance the permeability of the blood–brain barrier and facilitate drug transport to the brain [3,4]. It is also known for its antibacterial, antinociceptive, and therapeutic properties [5,6,7]. As borneol is safe and exhibits low aqueous solubility, it has potential as a matrix-forming component in ISGs.

One promising pharmaceutical application of ISG is to deliver antibacterial drugs for localized periodontitis treatment [8]. The emerging role of ISG is its alteration from injectable drug solutions into gels or matrices after solvent exchange due to exposure the aqueous phase, which can prolong drug release in the periodontal pocket [8,9]. Therefore, the organic solvent in the drug solution should be miscible with the aqueous phase and promptly diffuse from the ISG to an environmental aqueous phase, such as crevicular fluid in a periodontal pocket [10]. The matrix-forming agents are typically aqueous insoluble but are soluble in organic solvent [9]. The antimicrobial agents such as doxycycline hyclate and minocycline HCl are practically loaded in ISG for eradicating the pathogens of periodontitis in which the major anaerobic bacteria include *Aggregatibacter actinomycetemcomitans*, *Porphyromonas gingivalis*, and *Bacteroides forsythus* [10,11,12,13]. Atridox^®^ is a US-FDA approved commercial ISG of 10% doxycycline hyclate in 33.03% poly(D,L-lactide) (PLA) and 56.97% *N*-methyl-2-pyrrolidone (NMP) (Figure 1B) that can control drug release for 7 days [14]. Some aqueous-insoluble matrix-forming agents have been currently used as gelling or matrix-forming excipients of ISGs for periodontitis treatment, such as poly(lactic-co-glycolic acid) or PLGA, saturated fatty acids, eudragit RS, rosin, bleached shellac, and natural resins [15,16,17,18,19,20]. Given its safety, aqueous insolubility, and antimicrobial activities, therefore, borneol is interesting as a gelling or matrix-forming component of ISG. Moreover, it is an inexpensive hydrophobic material with numerous useful, non-toxic, and biocompatible properties.

Lincomycin HCl, a lincosamide bacteriostatic antibiotic (Figure 1C), contains a unique amino acid (propylhygric acid) linked by a peptide bond to a 6-amino-6,8-dideoxy-1-thio-D55 erythro-α-D-galactopyranoside sugar moiety [21,22,23]. Its mechanism of action involves binding to the 50S ribosomal subunit, thereby inhibiting protein synthesis in susceptible microorganisms [23,24]. Lincomycin HCl exhibits high activity against Gram-positive microorganisms and is commonly used to treat severe bacterial infections, such as sepsis, osteomyelitis, septic endocarditis, pneumonia, pulmonary abscess, infected wounds, and purulent meningitis [24]. It is particularly effective in the treatment of acute and chronic bone infections and soft-tissue infections in the oral and facial regions [23,25]. The antibiotic has shown susceptibility against anaerobic bacteria found in the oral cavity, including *P. gingivalis* [25,26]. Various drug delivery systems have been developed to achieve controlled release of lincomycin HCl, such as albumin nanoparticles and hydrogels for topical applications on wounds [27,28,29]. Recently, a borneol-based in situ forming matrix loaded with vancomycin HCl was fabricated with different matrix concentrations, demonstrating sustained drug release with 40% borneol in dimethyl sulfoxide [30]. However, no previous study has investigated the influence of drug concentration on the physicochemical properties of ISGs. Considering its susceptibility against anaerobic bacteria, lincomycin HCl-loaded ISGs have the potential to serve as an alternative antimicrobial delivery system for localized injection in periodontitis treatment. Therefore, it is intriguing to explore lincomycin HCl-loaded ISGs as a potential dosage form for the treatment of periodontitis.

In this study, we investigated drug-free and different concentrations of lincomycin HCl-loaded borneol-based ISGs for their physicochemical properties, including density, viscosity, surface tension, contact angle, mechanical properties, matrix formation, and drug release. We also evaluated their antimicrobial activities against *Staphylococcus aureus* ATCC 25923, *Escherichia coli* ATCC 8739, *Candida albicans* ATCC 10231, and *P. gingivalis* ATCC 33277.

## 2. Results and Discussion

### 2.1. Physicochemical Properties

#### 2.1.1. Physical Appearance, Density, and Viscosity

The drug-free and lincomycin HCl-loaded borneol-based ISGs were prepared into clear solutions by mixing for a short period using N-methyl-2-pyrrolidone (NMP) as a solvent. Increasing the concentration of lincomycin HCl turned its color from a colorless to a clear yellowish owing to the yellowish characteristic of the drug. The density values of the drug-free and lincomycin HCl-loaded borneol-based ISGs were significantly (*p* < 0.05) less than that of NMP (Table 1). The addition of borneol significantly (*p* < 0.05) decreased the density of the systems due to the low-density characteristic of borneol as reported in the literature as 1.01 g/cm^3^ [31]. Additional loading of lincomycin HCl increased the density of the ISGs; nevertheless, the density of NBL7.5 was slightly less than NMP. Meanwhile, the density values of other formulations were significantly less than that of NMP (*p* < 0.05). Typically, the density value of a mixture is the summation of the characteristic of each intact compound under the mole fraction ratio [32]. Overall, the density of the ISGs was higher than that of the aqueous phase, indicating that they would settle gradually into the crevicular fluid in the periodontal pocket.

The viscosity of the ISGs was higher compared to NMP, and it increased with the addition of borneol and lincomycin HCl, as shown in Table 1. The low viscosity was attributed to the simple structure and small molecular size of borneol, which reduced molecular interactions with NMP. This low viscosity and easy injectability are advantageous for in situ forming systems intended for localized drug delivery. Moreover, this was more beneficial than other viscous polymer-based in situ forming systems. The notably high viscosity of resin and polymer based ISGs has been reported previously [10,18,20]. The prepared ISG solutions containing 25% *w*/*w* ethyl cellulose, 35% *w*/*w* bleach shellac, and 40% *w*/*w* Eudragit RS were too viscous to flow [33]. Owing to comprising unsophisticated molecules, the ISGs with high loaded borneol or short chain saturated fatty acids exhibited markedly low viscosity and easy injectability [16,34,35,36].

#### 2.1.2. Surface Tension and Contact Angle

Ordinarily, surface tension is the property of a liquid’s surface film caused by the cohesive molecular attraction, which tends to minimize surface area [37]. The surface tension value of NB40 was significantly (*p* < 0.05) higher than those of NMP and the drug-loaded ISGs, as shown in Table 1. The addition of borneol seemed to enhance the cohesive force of NMP that might be due to hydrogen bonding. However, a decrease in the surface tension of the binary mixture between borneol–dimethyl sulfoxide was found when increasing borneol loading, and this had a saturated effect when the borneol concentration reached to 30% *w*/*w* [30]. The decreased surface tension of the ISGs is beneficial, as it promotes better spreadability and attachment to the target site, facilitating their application in the periodontal pocket.

Typically, the contact angle is the angle between a tangent of liquid drop and solid surfaces. The contact angles of NMP and ISGs on a glass slide, agarose gel, and paraffin surfaces are presented in Table 1. The contact angle of NMP on the glass slide was significantly (*p* < 0.05) higher than those of NB40 and drug-loaded ISGs, respectively, indicating that the addition of both borneol and lincomycin decreased the hydrophilicity of the formulation and declined the contact angle. For borneol, it is a lipophilic bicyclic monoterpene alcohol (log K_o/w_ = 2.85) [38]. The enhancement of viscosity owing to the higher drug loading decreased the speadability and slightly enhanced the contact angle on the glass surface. NMP was well spread on the agarose gel surface because of its hydrophilic characteristic that can be miscible with the aqueous phase. NB40 showed a significantly less contact angle than the drug-loaded ISGs, whereas its contact angle was significantly (*p* < 0.05) greater than that of NMP. The alteration from liquids into gels or ISG matrices was due to the solvent exchange after contacting with the aqueous agarose gel that retarded the spreadability of NB40 and drug-loaded ISGs. Nevertheless, increasing the drug concentration tended to decrease the contact angle due to greater hydrophilicity of the salt form of this drug on the agarose gel. By comparison, the rather high contact angle was evident of NMP and ISGs on the paraffin surface, indicating their hydrophilic manner on the paraffin’s hydrophobic surface. NMP, molecular weight of 99.13 daltons, is a very strong solubilizing agent with a log P of −0.38 and has low viscosity and high affinity with water [39,40,41]. It is normally used as a solubilizing excipient in several medications because it has a low acute toxicity via oral, dermal, and inhalation routes of administration [40,42]. The obtained contact angle values of all test specimens in this study were less than 90°, indicating their good wettability [43]. The sufficient wettability should be addressed for the effective adhesiveness between ISG and the surface to prohibit undesirable slippage of the transformed ISG from the target site such of the periodontal pocket [44].

#### 2.1.3. Water Tolerance

The initial phase separation of a hydrophobic compound such as borneol from the ISGs by NMP-solvent exchange could be detected from its turbidity when titrated ISGs with distilled water [36]. Less water tolerance indicates the lower amount of aqueous phase that induces a phase alteration or a lesser capacity of ISG to endure water. The water tolerances of drug free and drug-loaded borneol-based ISGs are presented in Table 1. Increasing the lincomycin HCl content in ISGs tended to decrease the water tolerance notably, signifying more aqueous sensitivity or ease for phase separation. In the case of higher drug loading, the solvent was not sufficient to dissolve borneol whenever the NMP diffused out, and water from the environment moved into the system. Moreover, the gradual addition of water into the ISG system also enhanced the system’s polarity because water has higher polarity than NMP in ISGs. The dielectric constant values of NMP and water are 32 and 78.54, respectively, at 20 °C [45]. The diminishing water tolerance value was also reported for fatty acid-based ISGs with higher fatty acids amount [34,35,36]. By comparison, the water tolerances of all ISGs at body temperature were higher than those at room temperature (Table 1). This result corresponds with the thermodynamic of endothermic solubility of a crystalline solute in liquid solvent, that its solubility proportionally increases by an elevation in temperature or entropy of the system [46]. In this respect, the alteration of lincomycin HCl-loaded borneol-based ISGs during solvent exchange in the periodontal pocket at body temperature should be greater than at room temperature. Nonetheless, the tiny amount of this injectable delivery system could expose and encompass crevicular fluid in the periodontal pocket to induce phase separation. It is important to note that the water tolerances of the ISGs were less than 14% *w*/*w* (Table 1), indicating that the system could easily reach its saturation point due to the incremental hydrophilicity of the system and the low aqueous solubility of the matrix-forming agent [36]. Therefore, careful consideration of the physicochemical properties of the components and temperature is necessary to ensure proper solvent exchange and in situ gel formation.

#### 2.1.4. In Situ Gel Formation

**Figure 2:**
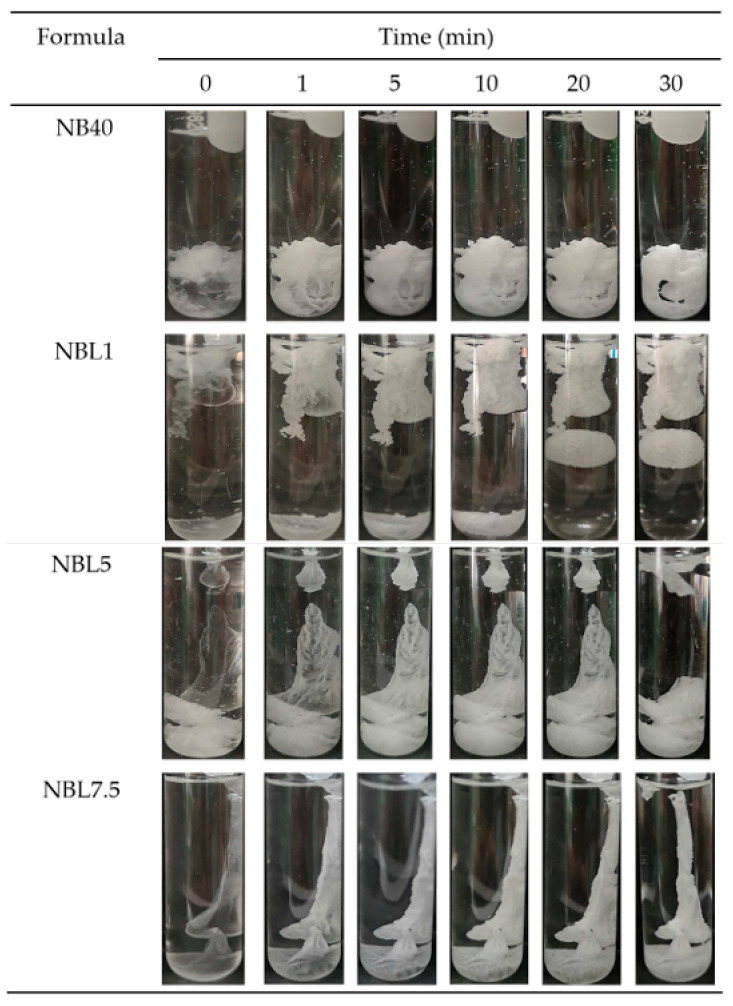
In vitro matrix formation behaviors of lincomycin HCl-loaded borneol-based ISGs in PBS pH 6.8.

**Figure 3:**
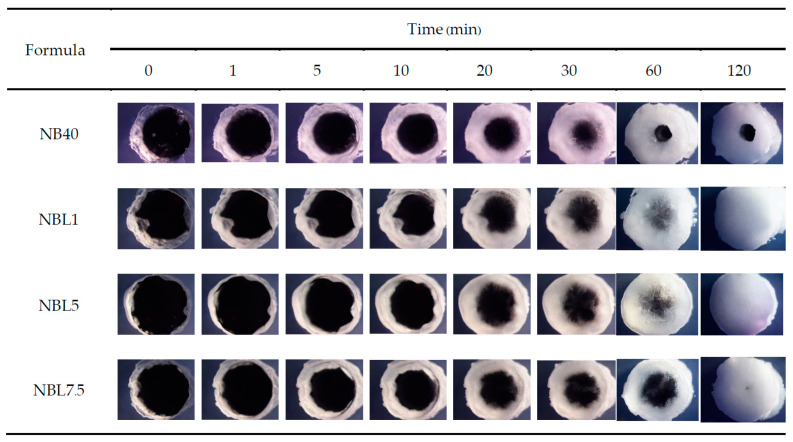
Cross-sectional view of gel formation behaviors of lincomycin HCl-loaded borneol-based ISGs in agarose wells under a stereo microscope at a magnification of 12×.

The phase transitions from the ISG solutions into gels or matrices after being injected into PBS pH 6.8 and agarose wells are depicted in Figure 2 and Figure 3, respectively. They suddenly changed after exposure to PBS into a cloudy mass, with some transparent gels remaining at the initial phase and gradually turning into cloudier matrices over time (Figure 2). NB40 rapidly agglomerated into an opaque mass and settled into the test-tube faster than NBL1, NBL5, and NBL 7.5, respectively, corresponding to their density values as previously described. Additional loading of lincomycin HCl slightly retarded the gel formation, owing to an increase in the hydrophilicity of the salt form of this drug. This gel formation was also evident and confirmed when using an agarose well instead of PBS (Figure 3). The cross-sectional view of gel formation in the agarose wells revealed the invasion of borneol matrix from the rim into the center of the agarose hole over time. Incomplete gel formation of NB40 in agarose wells was found, whereas drug-loaded ISGs revealed the complete gel formation within 120 min. The addition of lincomycin HCl could stimulate solvent exchange in the late state due to the hydrophilicity of drug in salt form, whereas the hydrophobic borneol matrix from NB40 acted as the barrier for the solvent exchange process and subsequently reduced the matrix formation. However, increased drug amount slightly delayed the gel formation that is similar to the result as previously mentioned in PBS pH 6.8. Nonetheless, the incorporation of 1% vancomycin HCl did not influence the rate of borneol matrix formation, which might be due to the minute amount of this drug loading, whereas a higher borneol concentration promoted a denser matrix to prolong the drug’s release [30]. The fatty matrix formation of lauric acid-based ISG occurred when it contacted the simulated crevicular fluid, changed from the solution state into a semisolid, and then to a solid-like state over time [16,35]. Gel formation in the ISG system relies on solvent exchange, in which there is a gradual phase inversion of a dissolved matrix forming agent into a gel state and finally into a solid matrix while its solvent diffuses outwardly and the aqueous phase from the environment moves as a replacement [36,39]. Even though the water tolerance of the ISGs was reduced by the addition of a drug, the rate of the gel formation seemed unrelated to water tolerance because adding a drug increased both the density and viscosity (as shown in Table 1) of ISGs and slightly delayed the formulation’s solvent exchange. Particularly, patient compliance from the sufficient phase alteration after injection by a dentist should be considered in the periodontal treatment process [47]. The large surface area on the small amount of ISG dosage form applied into a tiny periodontal pocket could expose crevicular fluid and be sufficient to induce its phase inversion.

#### 2.1.5. Mechanical Properties

**Figure 4:**
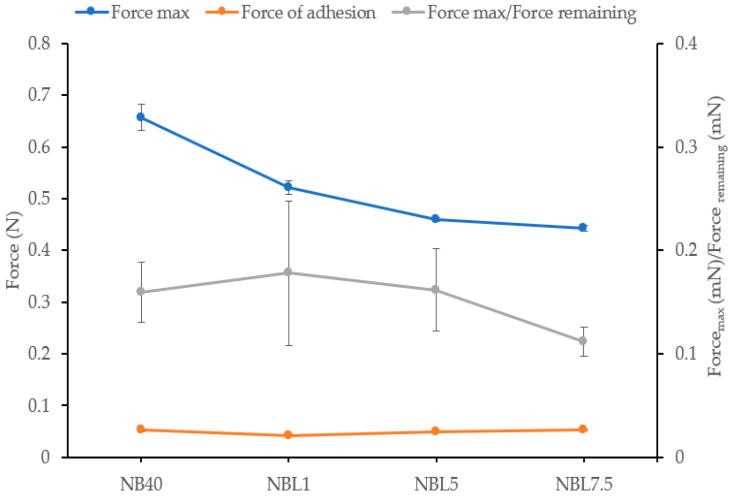
Mechanical properties of lincomycin HCl-loaded borneol-based ISGs after phase alteration for 24 h (*n* = 3).

The mechanical properties of ISGs play a crucial role in their ability to undergo phase inversion, resist jaw motion, and maintain their shape within the periodontal pocket [48]. To evaluate these properties, the ISGs were set in agarose wells to complete the phase alteration, and a texture analyzer was used to measure the gel’s hardness and the ratio of F_remaining_/F_max_ deformation. The F_remaining_/F_max_ deformation ratio serves as an indicator of plastic deformation (low value) or elasticity (high value) [49,50,51]. An analysis of Figure 4 revealed that the Fmax of the transformed matrix decreased with increasing drug loading, which correlated with the observed slower gel formation at higher drug concentrations. All ISG formulations exhibited a relatively low F_remaining_/F_max_ deformation ratio (<1), indicating a greater degree of plasticity than elasticity. This suggests that the ISGs possess favorable properties for retention and easy adaptation to the specific shape of the gum cavity in patients [49,50]. Notably, the F_remaining_/F_max_ deformation ratios were similar for ISGs with different drug concentrations, highlighting their consistent adhesive quality to the target site, which helps prevent the dosage form from slipping out of the periodontal pocket [49].

#### 2.1.6. Microscopic Interface Interaction

**Figure 5:**
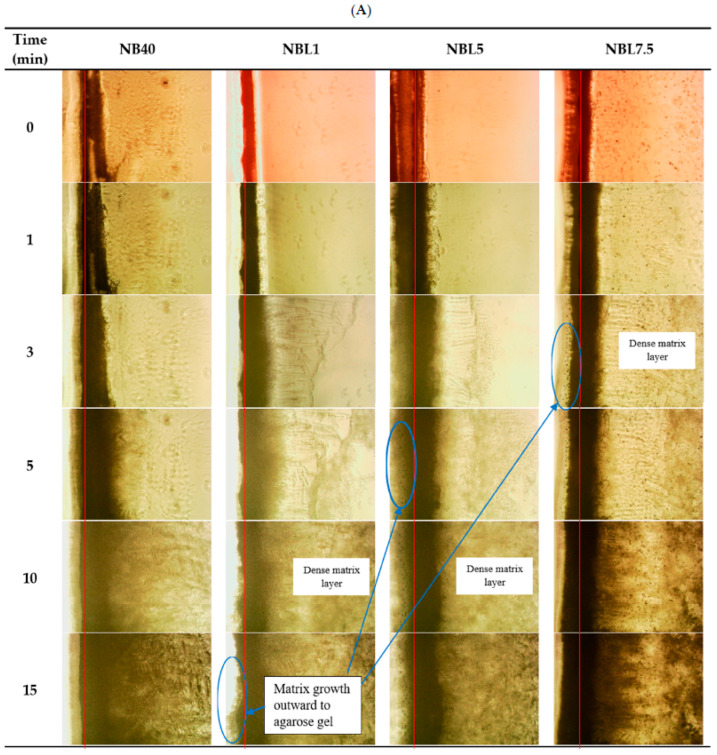

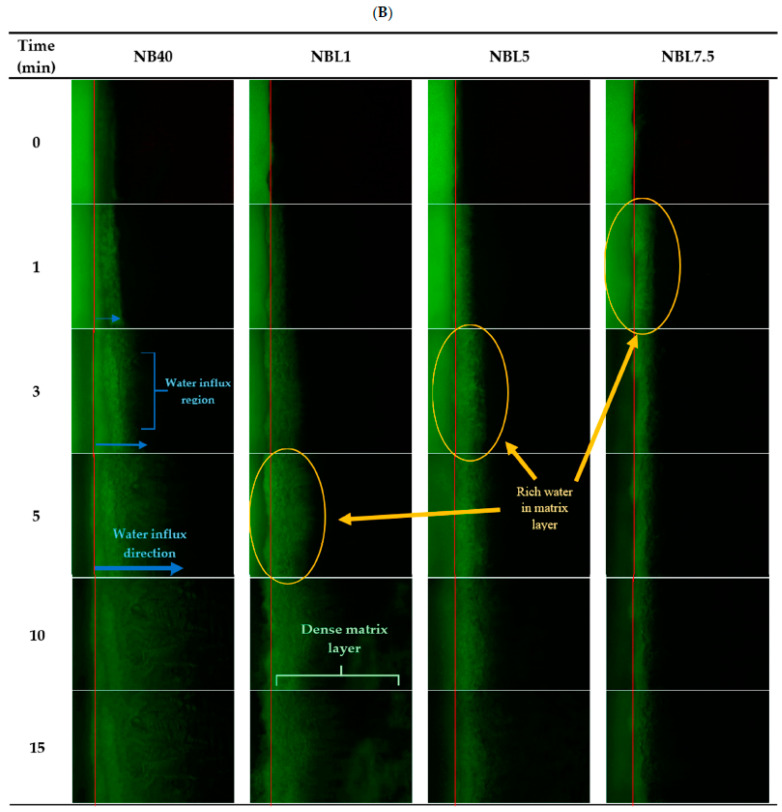
Matrix formation at the interface of the aqueous phase and ISGs under visible light (**A**) and fluorescence light (**B**) at 100× magnification under an inverted microscope. (The red line is the interface between agarose (left side) and formula (right side).)

The microscopic interface interaction between the ISGs and agarose gel was examined using natural light, as shown in Figure 5. This analysis aimed to understand the mechanism of phase transformation near the target site, which contains aqueous fluid [34,35,36]. The red line in the figure represents the initial boundary between the agarose gel (left) and the ISG formulation (right). In the case of NB40, the growth of the borneol matrix was rapid, resulting in a homogeneous, dark, and dense matrix mass that moved forward into the formulation faster compared to lincomycin HCl-loaded ISGs (Figure 5A). Additionally, a denser layer of small borneol crystals was observed separate from the matrix mass. This indicates that the increased solvent exchange, facilitated by water invading the formulation, promoted the phase inversion of the dense liquid ISG into gels and subsequently into the matrix mass after most of the solvent was removed [30,35]. The growth of the matrix continued to occur, involving nucleation and supersaturation-induced crystallization [36,52]. The growth of the borneol-based in situ forming matrices at the interface with the agarose rim changed rapidly with an increase in borneol concentration in DMSO [30]. In the case of lincomycin HCl-loaded ISGs, heterogeneous thinner matrices were observed, along with some breakage of the borneol crystal layer, consistent with the slow gel formation and low gel hardness mentioned previously in Section 2.1.4 and Section 2.1.5, respectively. The higher viscosity of ISGs with high drug concentrations could hinder solvent exchange and impede the matrix formation process of borneol. Interestingly, some borneol matrices also penetrated the agarose gel, indicating that small borneol molecules, along with NMP, could penetrate the agarose gel to a limited extent and precipitate due to their insolubility upon exposure to the higher polarity of the inner agarose’s water phase. In traditional Chinese medicine (TCM), borneol is believed to guide other active compounds to target tissues or organs, particularly the brain, due to its enhanced permeability of the blood–brain barrier [4]. Therefore, its small hydrophobic molecule promotes penetration, and increased drug loading prolongs penetration into the borneol matrix, as some dissolved borneol molecules diffuse through these heterogeneous matrices into deeper agarose and undergo crystallization.

The diffusion of water was tracked using a fluorescent probe and sodium fluorescein from the agarose gel (left) into the ISG formulations (right) under a fluorescent microscope (Figure 5B). The red line represents the interface between the agarose and the ISG formulation. Sodium fluorescein is an orange-red, odorless powder that is freely soluble in water and sparingly soluble in alcohol [52]. It absorbs blue light with peak excitation occurring at wavelengths between 465 and 490 nm, and the resulting fluorescence occurs at yellow-green wavelengths of 520–530 nm [53]. In NMP, sodium fluorescein does not exhibit fluorescence, hence the black background in the images. However, when dissolved in a medium containing a high amount of water, it exhibits a yellow-green color. The decreasing intensity of the green color in the agarose gel over time (Figure 5B) is attributed to the diffusion of sodium fluorescein and water into the inner formulation. This water diffusion stimulates the formation of a borneol matrix, which appears as an opaque band at the interface and expands into the formulation over time. Similarly, some borneol matrices penetrate into the agarose gel, consistent with the earlier observations under a stereoscope (Figure 5A). The broader fluorescent color of NB40 and the dense opaque band of the borneol matrix indicate a more efficient matrix formation compared to the drug-loaded ISGs. The green color tracking represents the region of water influx resulting from the diffusion of sodium fluorescein in the water phase. The green color of sodium fluorescein in PBS diffuses into the rosin-based ISG, and the presence of PBS induces matrix alteration through phase separation, leading to rosin precipitation [17]. The gradual movement of sodium fluorescein with sufficient water from the agarose into the formulation results in a brighter green color. Thus, inspecting the interface boundary and tracking the movement of sodium fluorescein provides valuable information about the ability of water to move and the behavior of matrix formation through the phase inversion of ISGs.

#### 2.1.7. Drug Content and In Vitro Drug Release

**Figure 6:**
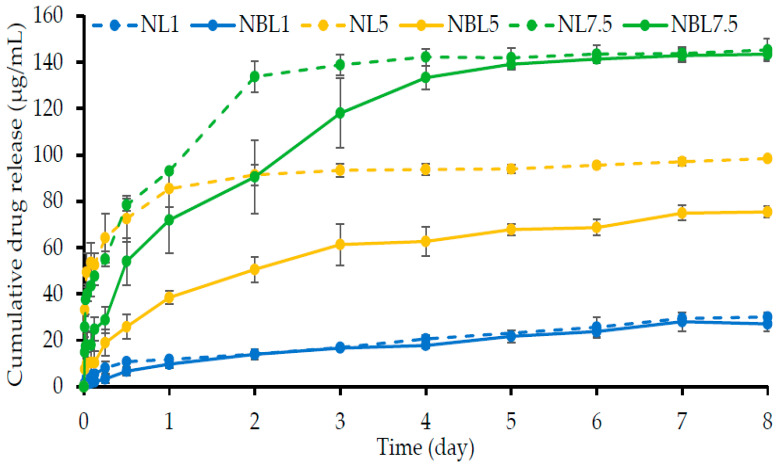
Lincomycin HCl release from borneol-based ISGs in PBS pH 6.8 using the cup method (*n* = 6).

The lincomycin HCl content in the NL1, NL5, NL7.5, NBL1, NBL5, and NBL7.5 formulations was found to be 99.01 ± 0.84, 101.77 ± 0.26, 100.82 ± 0.16, 100.83 ± 0.32, 99.36 ± 0.42, and 98.72 ± 0.72, respectively. These formulations were prepared using a simple mixing process, resulting in lincomycin HCl content close to 100% of its intended addition. Figure 6 shows the drug release profiles of lincomycin HCl from the borneol-based ISGs in PBS pH 6.8. The borneol concentration for these ISG preparations was 40% *w*/*w* because it was the highest concentration for incorporating lincomycin HCl. In addition, the borneol-based in situ forming matrix was recently fabricated to load vancomycin HCl by varying the concentration of the matrix-forming agent. This demonstrated that 40% *w*/*w* borneol in dimethyl sulfoxide was appropriate for sustained drug release [30]. The insufficient transformation rate into matrix was mentioned for the borneol-ISG systems at lower 40% *w*/*w*, in which they might escape by flowing as a liquid from the periodontal pocket before matrix formation. However, the impact of drug concentration on physicochemical properties of the borneol-based ISG has not been reported. NMP was selected as a solvent because of its low toxicity (oral and dermal LD_50_ in rodents of 3600–7700 mg/kg) and its utilization in a commercial dosage form such as Atridox^®^ for periodontitis treatment [14,54]. The drug release behavior of these lincomycin HCl-loaded borneol-based ISGs was conducted by the cup method to mimic the surrounding environment of the periodontal pocket [14,15]. Moreover, several research publications have employed this technique to follow the drug release of ISG formulations [55,56,57]. The lincomycin HCl release from the control groups, including NL1, NL5, and NL7.5, was greater than those of their ISG formulations (Figure 6). All control groups reached the plateau state of drug release within two days; meanwhile, those of the drug-loaded borneol-based ISGs were gradually liberated over 8 days. Although, the release profiles of borneol-free and borneol-based solutions containing 1% *w*/*w* lincomycin HCl were similar in that they had a slightly lesser amount of released drug. The commercial product Atridox^®^, containing doxycycline hyclate, achieved sustained drug release for seven days [14,54]. Thus, the lincomycin HCl-loaded borneol-based in situ forming matrices demonstrated the ability to control drug release for at least eight days. After an initial burst release from drug deposited on the surface of the in situ forming matrices, the drug was controlled to release more slowly (Figure 6). Usually, the hydrophobic matrix, such as solid borneol mass, following complete phase alteration, should impede drug liberation more efficiently than its initial liquid and gel states [58]. These characteristics make the borneol-based in situ forming matrices promising for controlled release applications, offering enhanced patient compliance by reducing the frequency of drug administration [9,57]. If more patient-specific factors can be identified, such as the state of periodontitis, disease severity, shape of the patient’s periodontal pockets, or hereditary traits that affect how drugs respond or the growth of pathogens, these parameters might be used to determine the properties and components of the formula to suit each patient. Therefore, the designed ISG containing different drug loading, rate of matrix formation, and drug release characteristics could be personalized to specific patients’ needs to insure the best therapeutic response with the highest efficacy and safety margin [59].

The release data were fitted to different mathematical models, and the degree of goodness-of-fit was evaluated using the coefficient of determination (R2), Akaike information criterion (AIC), and model selection criterion (MSC) [60]. The high values of R^2^ and MSC together with the low value of AIC are signified to indicate the best fit or high degree of goodness of curve fitting [60]. The estimated parameters obtained after fitting release data with zero-order, first-order, Higuchi’s, Korsmeyer–Peppas’s, and Peppas–Sahlin’s equations are also presented in Table 2. The release profiles of most formulations showed a high degree of goodness-of-fit with the Peppas–Sahlin’s equation, indicated by high R2 values close to 1.0. The estimated parameters obtained from the curve fitting provided insights into the release mechanisms. Higher k1 values suggested that diffusion was the main mechanism of drug release, while higher k2 values indicated dominant polymer relaxation or heterogeneous erosion [60,61]. Negative k values could typically be observed and indicated as one term in the model for compensation of producing the best fit of data; nevertheless, this should not be included when interpreting the release mechanism comparing the k value [60]. By comparison, most of the drug release from control groups without borneol addition followed the first-order release kinetic (Table 2) in which the drug was freely liberated from the solution with concentration dependence. Fickian diffusion was the predominant release mechanism observed in the NBL1 and NBL5 formulations, as evidenced by their higher k1 values and good fit with Higuchi’s equation (Table 2). The release mechanism of imatinib mesylate from the rosin-based ISG [62] and doxycycline hyclate from the lime peel oil-loaded rosin-based formulations [63] mainly obeyed Fickian diffusion. Generally, a Fickian diffusion-driven matrix release mechanism is related to a concentration gradient, diffusion distance, and swelling degree [64]. The release mechanism of NBL7.5 appeared to be closer to first-order release kinetics but also showed a good fit with Higuchi’s equation and a higher k1 value in the Peppas–Sahlin’s equation, suggesting a Fickian diffusion mechanism similar to NBL1 and NBL5. The initial burst release from the in situ forming matrices was attributed to drug deposited near the gel surface, while the subsequent transition from the gel state to the solid-like borneol matrix effectively retarded drug diffusion, leading to prolonged release.

The sustained release of lincomycin HCl from the borneol-based ISG is particularly relevant in inhibiting microbial growth, as lincomycin has been reported to be effective against anaerobic bacteria, including *P. gingivalis*, commonly found in the oral cavity [25,26]. The attempts to characterize the topography of borneol matrices after a dissolution test were also undertaken using scanning electron microscopy, though its sublimable manner made this mission impossible. This unique sublimable property of borneol has been applied to fabricate highly porous, low-density, and non-effervescent floating tablets [65]. Besides, borneol was reported as an enhancer for drug absorption in the gastrointestinal tract and drug distribution to the brain [66]. It is effective and safe to use as an ocular penetration enhancer by increasing transcorneal penetration of both hydrophilic and lipophilic compounds [67]. Therefore, the borneol-based ISG might promote some antimicrobial drug penetration through periodontal tissue to eradicate pathogens that have invaded in the case of aggressive periodontitis. Moreover, the delivery system of this developed ISG should cause less drug resistance and diminish the drug’s side effects [68]. Meanwhile, it could deliver the drug at the infectious site locally with apparent high drug loading to completely eliminate periodontal pathogens from the chronic periodontal pockets [69]. The minimum inhibitory concentration (MIC) value of lincomycin against *P*. *gingivalis* was 16–32 µg/mL as reported by Heung-Sik and Hyung-keun [26]; therefore, the drug release from NBL5 and NBL7.5 could achieve the MIC drug level on the first day and sustain drug release above the MIC over 8 days and, thus, inhibit microbial growth.

### 2.2. Antimicrobial Activities

**Figure 7:**
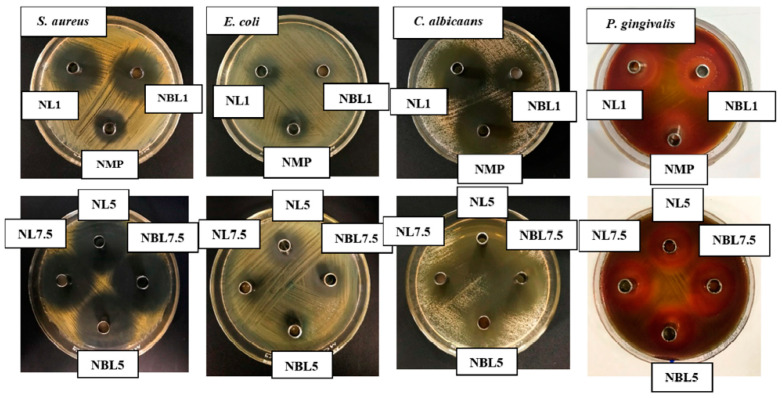
Inhibition zone of lincomycin HCl-loaded borneol-based ISG systems against *S. aureus* ATCC 25923, *E. coli* ATCC 8739, *C. albicans* ATCC 10231, and *P. gingivalis* ATCC 33277.

The antimicrobial activities of the developed ISG formula, compared with its solvent and drug solutions, were evaluated against *S. aureus* ATCC 25923, *E. coli* ATCC 8739, *C. albicans* ATCC 10231, and *P. gingivalis* ATCC 33277, which are commonly associated with periodontitis [12,70,71]. All lincomycin HCl-loaded borneol-based ISGs inhibited the growth of all test microbes (Table 3, Figure 7) in a dose-dependent manner. However, their inhibition zone diameters were smaller than those of their drug solutions without borneol. This result clearly demonstrated that the borneol matrix formed due to the solvent exchange induced by water from the agar media, which retarded the diffusion of the drug.

Significantly larger inhibition zones were observed for all samples against *S. aureus* ATCC 25923, *P. gingivalis* ATCC 33277, and *E. coli* ATCC 8739, indicating the efficient inhibition of their growth by the developed ISGs. However, NBL1 showed a significantly smaller inhibition zone diameter than NL1 (*p* < 0.05) (Table 3). Practically, lincomycin is a reserve drug for infections caused by strains of *Staphylococci* and other *Gram*-positive microorganisms that are resistant to penicillin or for patients allergic to penicillin [24]. In dentistry, lincomycin is effective in treating acute and chronic bone infections and soft-tissue facial infections [23,25], and it is susceptible to an anaerobe bacterium including *P. gingivalis* [25,26].

For *C. albicans* ATCC 10231, NMP exhibited dominant antifungal activity, while lincomycin HCl also showed some activity. NMP exhibited a large inhibition zone, although smaller than those of NL1, NL5, and NL7.5 solutions (Table 3). Previous studies have reported the good inhibition of intact NMP and NMP-loaded thermosensitive gels against *C. albicans* and bacteria [57,72,73]. Thus, these ISGs and their drug solutions also inhibited these fungus and bacteria. The drug-loaded ISGs presented a significantly (*p* < 0.05) smaller inhibition zone of antifungal activity than their drug solutions because the borneol matrix formation retarded drug diffusion. NMP showed significantly smaller inhibition zone diameter against *S. aureus* ATCC 25923 and *P. gingivalis* ATCC 33277 than drug-loaded ISGs and drug solution. This smaller inhibition zone diameter of some borneol-based ISGs can be attributed to the retardation of NMP diffusion by the borneol matrix, consistent with the drug release mechanism described previously. The ISG matrices obtained from fatty acids [34,35,36], natural resins [17,56,62,63], oligosaccharide [57], and other polymers [33,55] have been reported to impede drug diffusion and decrease the diameter of the microbial inhibition zone while enabling sustained drug release.

Borneol has been used in traditional Chinese medicine for over 1600 years [74,75]. Chinese medicine preparations such as Angong Niuhuang pills and Xingnaojing injection, which contain borneol, are widely used in the clinical treatment of stroke [76,77]. Borneol has also demonstrated anti-fibrotic activity by possibly inhibiting fibroblast mitosis, collagen production, and TIMP-1 production, making it safe for the treatment of oral submucous fibrosis [78]. Borneol has low toxicity and has been tested for skin irritation in human volunteers and mice [3]. In immune cells, borneol at concentrations of 0.5 and 5 µg/mL did not induce significant toxicity and even increased cell viability [79]. Therefore, the use of borneol as a matrix-forming agent in ISGs is of interest due to its safety, efficient antimicrobial activities, and ability to modulate drug release. The oral absorption bioavailability of lincomycin HCl is 20 to 30% in the fast state and decreases in the fed state [80]. The high local drug concentration with decreasing undesirable side effects was achieved by delivery to the periodontal pocket. The release of lincomycin HCl from chitosan films for periodontitis treatment showed the sustained release without any burst effect for 5 consecutive days [81].

The lincomycin HCl-loaded borneol-based ISGs NBL5 and NBL7.5 released the drug in amounts reaching the MIC value against *P. gingivalis* on the first day and sustained drug release above the MIC value for over 8 days, considering the previously reported MIC value of lincomycin against *P. gingivalis* as 16–32 µg/mL [26]. While there are safety data available for borneol’s medical applications, further clinical experiments are required to investigate the efficacy and safety of these lincomycin HCl-loaded borneol-based ISG formulations. The treatment of osteomyelitis and soft tissue infections using lincomycin has been reported for its implications in the field of dentistry because of its effective ability to penetrate bone and severely infected tissue [82]. Moreover, lincomycin incorporated in a tricalcium phosphate carrier could accelerate wound healing and reduce complications, such as alveolar periostitis, pain, trismus, and atrophy of the alveolar process, after surgical extraction of impacted third molars [83]. Lincomycin HCl-loaded chitosan and chitosan–gelatin matrices have also been reported for inhibiting local infections of the oral cavity [84]. In addition to intravenous or intramuscular delivery, the use of ISGs could be an alternative treatment for site-specific microbial infections, providing high local drug concentrations while minimizing undesirable side effects. Typically, the ISG for periodontal pocket drug delivery is a freshly prepared dosage form. The constituted product of Atridox^®^ for periodontitis treatment is a viscous liquid after freshly prepared with a concentration of 10% of doxycycline hyclate [14]. Upon contact with the crevicular fluid, the liquid product solidifies and then allows for controlled release of a drug for a period of 7 days. Therefore, the instability of developed ISG should not be noticeable. The borneol-based matrix of an in situ forming system showed self-degradation via evaporation from our group investigation [85]. The borneol-based in situ forming system with phosphate buffer solution (PBS)-induced phase inversion was checked for its in vitro degradation of borneol matrix over time. The evaporation behavior data of the borneol-based in situ forming system is valuable for developing the self-degradable ISG for periodontitis. Whilst there are safety data on borneol’s medical applications as mentioned above, these lincomycin HCl-loaded borneol-based ISG formulations need to be further investigated through clinical experiments for their efficacy and safety.

## 3. Conclusions

In conclusion, this study successfully developed lincomycin HCl-loaded ISGs using 40% borneol as a matrix-forming agent and NMP as a solvent. The ISGs exhibited low viscosity and surface tension, making them easily injectable and spreadable, respectively. The gel formation process involved solvent exchange and the formation of a hydrophobic borneol matrix, which affected the water tolerance and gel properties of the formulations. The addition of lincomycin HCl influenced the solvent exchange process and reduced the matrix formation. The borneol matrix showed adaptable plasticity properties, conforming to the shape of a patient’s gum cavity. The diffusion studies using sodium fluorescein indicated the behavior of water and borneol matrix formation at the interface boundary. The lincomycin HCl-loaded ISGs exhibited thinner and heterogeneous borneol matrices, consistent with the gel formation and hardness results. The drug release mechanism followed Fickian diffusion, as evidenced by the fitting of Peppas–Sahlin’s equations and Higuchi’s equation. NBL5 and NBL7.5 formulations achieved the minimum inhibitory concentration (MIC) of lincomycin HCl on the first day and sustained drug release above MIC for over 8 days. The lincomycin HCl-loaded borneol-based ISGs showed efficient inhibition of the growth of *S. aureus* ATCC 25923, *E. coli* ATCC 8739, *C. albicans* ATCC 10231, and *P. gingivalis* ATCC 33277. Therefore, the 5% and 7.5% lincomycin HCl-loaded 40% borneol-based ISGs have potential as localized drug delivery systems for periodontitis. However, further investigation is needed to assess their efficacy and safety in clinical settings.

## 4. Materials and Methods

### 4.1. Materials

In this study, the following materials were used. Lincomycin HCl (Lot no. 2005012) was generously provided by NOVA Medicine Company Ltd., located in Pathumthani, Thailand. Borneol was procured from Chareorsuk-osod Herbal Shop in Nakhon Pathom, Thailand. NMP (Lot no. 144560-118, QReC, Auckland, New Zealand) served as the solvent for dissolving lincomycin HCl and borneol. Phosphate buffered solution (PBS) with a pH of 6.8 was prepared using sodium hydroxide (Lot no. AF310204) and potassium dihydrogen orthophosphate (Lot no. E23W60) obtained from Ajax Finechem in New South Wales, Australia. To create the agarose gel, 0.6% agarose (Lot no. H7014714) from Vivantis in Selangor Darul Ehsan, Malaysia, was dissolved in PBS with a pH of 6.8.

Tryptic Soy Agar (TSA) (Lot no. 7341698) and Tryptic Soy Broth (TSB) (Lot no. 8091999) from DifcoTM, based in Detroit, MI, USA, were utilized as the media for cultivating *S. aureus* ATCC 25923 and *E. coli* ATCC 8739, respectively. Sabouraud Dextrose Agar (SDA) (Lot no. 7312647) and Sabouraud Dextrose Broth (SDB) (Lot no. 6345690), also from DifcoTM in Detroit, MI, USA, were used as the media for *C. albicans* ATCC 10231. The sheep blood agar, provided by the Department of Medical Science, Ministry of Public Health in Thailand, was used as the media for *P. gingivalis* ATCC 33277 obtained from MicroBiologics Inc., located in St Cloud, MN, USA.

### 4.2. Preparation of Drug-Free and Lincomycin HCl-Loaded Borneol-ISG Solutions

To prepare the drug-free and lincomycin HCl-loaded borneol-based ISG solutions, various concentrations (1, 5, and 7.5% *w*/*w*) of lincomycin HCl were incorporated into ISG solutions containing 40% *w*/*w* borneol. NMP was used as the solvent for the preparation process. The mixtures were continuously stirred for 2.5 h using a magnetic stirrer until transparent solutions were achieved. As a control formulation, a solution of 40% *w*/*w* borneol dissolved in NMP (referred to as NB) was prepared. The components of the formulations are summarized in Table 4.

### 4.3. Physicochemical Study

#### 4.3.1. Density and Viscosities

The density of NMP and the ISG formulations was directly measured using a pycnometer (Densito 30PX, Mettler Toled Ltd., PortableLabTM, East Bunker Ct, Vernon Hills, IL, USA) (*n* = 3). The obtained density values for each formulation were used for subsequent calculations of the surface tension. Viscosity and shear stress of NMP and the ISG formulations were determined using a viscometer (Brookfield Engineering Laboratories Inc., Middleborough, MA, USA) with a CP-40 spindle (*n* = 3) at a temperature of 25 °C and a shear rate of 250 s-1. These measurements provided insights into the flow properties of the formulations.

#### 4.3.2. Surface Tension and Contact Angle

The contact angle values of NMP and the ISG formulations on various surfaces, including a glass plate, paraffin, and agarose gel, were measured using a goniometer (FTA 1000, First Ten Angstroms, Newark, CA, USA) with a pump-out rate of 1.9 µL/sec. The contact angle was recorded at a time point of 5 s (*n* = 3). Additionally, the surface tension of the ISG solutions was determined by measuring the pendant drop using the same goniometer under the same conditions (*n* = 3) for which the density value from Section 4.3.1 was used for this determination. These measurements provided information about the wetting behavior and interfacial properties of the formulations.

#### 4.3.3. Water Tolerance Test

The ability of the ISG formulations to tolerate water-induced phase separation was evaluated to assess their capacity to maintain a stable solution state when exposed to water. In this test, 20 μL of deionized water was gradually added using a micropipette to 2.5 g of the sample in a glass test tube. The mixture was then stirred using a vortex mixer until the clear ISG solutions turned cloudy due to phase separation of borneol. The experiment was conducted at temperatures of 25 °C and 37 °C. The water tolerance value was determined using the following Equation (1) (*n* = 3), indicating the amount of water that could be added before phase separation occurred.


(1) $$\%water\;tolerance=\frac{water\;amount(g)}{sample\;amount\left(g\right)+water\;amount(g)}\times 100\%$$

#### 4.3.4. Gel Formation Study

The transition of ISG solutions into gels or matrices was investigated through visual observation. The formulations were injected using a 1 mL syringe fitted with an 18-gauge needle into PBS (pH 6.8), and the changes were recorded at different time intervals (1, 5, 10, 15, and 30 min). Additionally, microscopic analysis of gel formation was conducted by adding 150 μL of ISG into a 300 μL hole (with a diameter of 7 mm) in agarose wells. The formation of gels or matrices was captured using a stereomicroscope (SZX10, Olympus Corp., Tokyo, Japan) at 0, 1, 5, 10, 20, and 30 min.

#### 4.3.5. Mechanical Properties

The mechanical properties of the lincomycin HCl-loaded borneol-ISGs were evaluated using a texture analyzer (TA.XT Plus, Stable Micro Systems Ltd., Godalming, UK). A volume of 150 μL of ISG was filled into agarose wells with a 300 μL hole (diameter of 7 mm) and allowed to undergo phase alteration for 72 h. Subsequently, a stainless probe attached to the texture analyzer was lowered into the prepared sample at a rate of 0.5 mm/s. The probe remained in position for 60 s, after which it was retracted at a speed of 10 mm/s. The maximum force (Fmax) exerted by the probe during penetration indicated the hardness of the sample, while the force recorded during the probe’s upward movement represented the adhesive force between the sample surface and the probe. The mechanical properties were expressed as the ratio of the remaining force to the maximum penetration force, where higher values indicated greater elasticity and lower values indicated higher plasticity [49]. The measurements were performed in triplicate.

#### 4.3.6. Interfacial Phenomena between Formulation and Aqueous Phase

To investigate the interfacial behavior between the formulations and the aqueous phase, plain agarose gel (0.6% *w*/*w*) and sodium fluorescein-loaded agarose gel (0.4 μg/mL) were prepared by dissolving them in PBS pH 6.8 at 60 °C. The agarose gels were cut at the edges, and 50 µL of the ISG formulations were carefully applied to the agarose rims. The phase alteration at the interface was observed using an inverted microscope (TE-2000U, Nikon, Kaw, Japan) under visible light or a blue (B2A) filter excited at 450–490 nm to visualize the green color of sodium fluorescein. Images were captured at 0, 1, 3, 5, 10, and 15 min to monitor the changes occurring at the interface.

#### 4.3.7. Drug Content and In Vitro Drug Release Studies

The lincomycin HCl content in the prepared samples was determined using high-performance liquid chromatography (HPLC) (Agilent 1260 Infinity, San Diego, CA, USA) at 264 nm with a C18 column (150 × 4.6 mm, 5 µm particle size, Dr.Maisch GmbH, Munich, Germany) (*n* = 6), following a standard curve analysis.

The release behavior of lincomycin HCl from the lincomycin HCl-loaded borneol-ISGs was studied using the porcelain cup method. The ISGs and control formulation (NB40) were weighed (0.3 g) and filled into cups using a micropipette on analytical balance. The cups were then carefully placed in glass bottles containing 80 mL of PBS pH 6.8. The release study was conducted at 37 °C using an incubator (Model NB-205, N-Biotek, Gyeonggi-do, Korea) with a rotational speed of 50 rpm. At specific time intervals, 3 mL of the release medium was sampled, and an equal volume of fresh PBS was replaced to maintain a sink condition. The concentration of lincomycin HCl in the sampled release medium was determined (*n* = 6) using HPLC (Agilent 1260 Infinity, San Diego, CA, USA) at 264 nm with a C18 column (150 × 4.6 mm, 5 µm particle size, Dr. Maisch GmbH, Munich, Germany). The mobile phase for drug analysis consisted of 75% acetonitrile and 25% phosphate buffer saline pH 6.8, which was filtered through a 0.45 µm membrane filter and sonicated for 30 min prior to use. The analysis was performed with an isocratic elution at a flow rate of 1 mL/min, and the column temperature was maintained at ambient temperature. A sample injection volume of 20 µL and a detection wavelength of 204 nm were used. The intact powder of lincomycin HCl was used as the standard, dissolved in the release medium to prepare the calibration curve, following the same method and drug analysis as described above.

The release kinetics of lincomycin HCl from borneol-based ISGs were evaluated by fitting the dissolution profile to different mathematical models, including zero order, first order, Higuchi’s, Korsmeyer–Peppas’s, and Sahlin–Peppas’s models. The DD-Solver software, an add-in program for Microsoft Excel (Redmond, WA, USA), written in Visual Basic applications, was used for the analysis of release kinetics. The *n*-value from the Korsmeyer–Peppas’s equation and the k1 and k2 values from Sahlin–Peppas’s equation were used to indicate the kinetic of drug release. The goodness of curve fitting was assessed by calculating R2, AIC, and MSC values.

#### 4.3.8. Antimicrobial Activities

The antibacterial and antifungal activities of the drug-free and lincomycin HCl-loaded borneol-ISG solutions were evaluated against standard microbial strains, including *S. aureus* ATCC 25923, *E. coli* ATCC 8739, *C. albicans* ATCC 10231, and *P. gingivalis* ATCC 33277. For bacteria, the inocula were incubated for 36 h in TSB, and the turbidity of the broth suspensions was calibrated using the 0.5 McFarland standard. The prepared broth suspensions of *S. aureus* ATCC 25923 and *E. coli* ATCC 8739 were swabbed and spread on TSA plates. As for *P. gingivalis* ATCC 33277, sheep blood agar was used for antimicrobial testing. The calibrated inoculum of *C. albicans* ATCC 10231 was swabbed and spread on SDA. Sterilized cylindrical cups were carefully placed on the surface of the swabbed agar. Then, 200 μL of the lincomycin HCl-loaded borneol-ISG solutions or drug-free ISG solutions were added to the cups. The plates were incubated at 37 °C for 24 h (72 h for anaerobic bacteria in an anaerobic incubator). After incubation, the diameter of the inhibition zones was measured using a standard ruler, and photographs were taken. The mean values and standard deviations were calculated and reported (*n* = 3). This process was repeated for each microbial strain to assess the antimicrobial activity of the formulations.

### 4.4. Statistical Analysis

The data obtained from the experiments are reported as mean values ± standard deviation (SD). To assess the differences between groups, a one-way analysis of variance (ANOVA) was performed. The statistical analysis was conducted using SPSS for Windows, version 11.5. A *p*-value of less than 0.05 was considered statistically significant.

## Figures and Tables

**Table 1:** Physical properties of lincomycin HCl-loaded borneol ISG formulations (*n* = 3).

Formula	Density (g/cm^3^)	Viscosity (cP)	Surface Tension (mN/m)	Contact Angle (Degree)			% Water Tolerance (% *w*/*w*)	
				Glass Slide	Agarose Gel	Paraffin	25 °C	37 °C
NMP	1.027 ± 0.001 ^a^	2.04 ± 0.13 ^b^	39.31 ± 0.28	31.08 ± 0.40 ^d^	7.12 ± 1.51	44.68 ± 2.15	-	-
NB40	1.010 ± 0.000 ^a^	3.76 ± 0.06	45.70 ± 0.25 ^c^	17.16 ± 1.20 ^d^	26.98 ± 1.64 ^e^	46.56 ± 0.53	12.76 ± 0.29	13.16 ± 0.00
NBL1	1.011 ± 0.001 ^a^	4.01 ± 0.01 ^b^	40.18 ± 0.05	9.15 ± 1.98	37.75 ± 1.60	45.82 ± 0.39	11.52 ± 0.36	13.16 ± 0.00
NBL5	1.019 ± 0.000 ^a^	5.51 ± 0.02 ^b^	40.64 ± 0.20	10.37 ± 0.46	35.01 ± 1.56	46.84 ± 0.93	9.39 ± 0.00	11.32 ± 0.00
NBL7.5	1.025 ± 0.000	6.78 ± 0.02 ^b^	40.12 ± 0.32	13.58 ± 0.38	32.54 ± 1.28	39.16 ± 1.08	8.29 ± 0.39	9.39 ± 0.65

The superscripts ^a–e^ in the columns represent a significant difference (*p* < 0.05) within the tested formulations.

**Table 2:** Degrees of goodness-of-fit and estimated parameters from drug release profile fittings of lincomycin HCl-loaded borneol-based ISGs in PBS pH 6.8 using the cup method.

Formulation	Modeling	Criteria for Model Selection			Kinetic Parameters		
		R^2^	AIC	MSC			
NL1	Zero order	0.7505	116.6260	1.1271	k_0_ = 14.408		
	First order	0.8812	106.1227	1.8774	k_1_ = 0.363		
	Higuchi’s	0.9615	90.1917	3.0153	k_H_ = 35.490		
	Korsmeyer–Peppas’s	0.9708	87.6678	3.1956	k_KP_ = 39.953	*n* = 0.423	
	Peppas–Sahlin’s	0.9393	49.8984	2.4443	k_1_ = 33.959	k_2_ = 6.109	m = 0.311
NL5	Zero order	1.3967	155.4462	−1.5498	k_0_ = 16.864		
	First order	0.7183	123.0316	0.6112	k_1_ = 6.485		
	Higuchi’s	0.0977	143.5405	−0.7561	k_H_ = 44.503		
	Korsmeyer–Peppas’s	0.9380	49.9655	2.6167	k_KP_ = 79.212	*n* = 0.173	
	Peppas–Sahlin’s	0.9488	49.4285	2.6764	k_1_ = 97.941	k_2_ = −17.041	m = 0.216
NL7.5	Zero order	0.1328	155.7117	−0.1901	k_0_ = 16.238		
	First order	0.8631	126.0518	1.6636	k_1_ = 1.397		
	Higuchi’s	0.7421	136.2893	1.0237	k_H_ = 41.861		
	Korsmeyer–Peppas’s	0.9515	48.4314	2.8900	k_KP_ = 64.463	*n* = 0.328	
	Peppas–Sahlin’s	0.9534	48.5188	2.8803	k_1_ = 38.209	k_2_ = 26.017	m = 0.247
NBL1	Zero order	0.8892	113.1104	1.9081	k_0_ = 12.892		
	First order	0.9700	90.2398	3.4328	k_1_ = 0.261		
	Higuchi’s	0.9834	84.4966	3.8157	k_H_ = 30.920		
	Korsmeyer–Peppas’s	0.9791	36.1348	3.6937	k_KP_ = 29.361	*n* = 0.527	
	Peppas-–-Sahlin’s	0.9777	36.8636	3.6026	k_1_ = 29.806	k_2_ = −0.508	m = 0.585
NBL5	Zero order	0.6336	125.7564	0.7413	k_0_ = 11.854		
	First order	0.8453	111.2304	1.7097	k_1_ = 0.249		
	Higuchi’s	0.9395	98.3918	2.5656	k_H_ = 29.375		
	Korsmeyer–Peppas’s	0.9512	55.5895	2.8777	k_KP_ = 35.368	*n* = 0.389	
	Peppas–Sahlin’s	0.9819	47.7392	3.6627	k_1_ = 37.226	k_2_ = −1.288	m = 0.547
NBL7.5	Zero order	0.6748	141.8234	0.9285	k_0_ = 15.467		
	First order	0.9573	109.5144	2.9478	k_1_ = 0.570		
	Higuchi’s	0.9477	111.4238	2.8284	k_H_ = 38.303		
	Korsmeyer–Peppas’s	0.9697	36.5037	3.2068	k_KP_ = 44.108	*n* = 0.495	
	Peppas–Sahlin’s	0.9730	35.8043	3.3067	k_1_ = 47.833	k_2_ = −2.585	m = 0.549

**Table 3:** Clear zone diameter of the lincomycin HCl-loaded borneol-based ISG systems against *S. aureus* ATCC 25923, *E. coli* ATCC 8739, *C. albicans* ATCC 10231, and *P. gingivalis* ATCC 33277 (*n* = 3).

Formula	Clear Zone Diameter (Mean ± SD)			
	*S. aureus* ATCC 25923	*E. coli* ATCC 8739	*C. albicans* ATCC 10231	*P. gingivalis* ATCC 33277
NMP	17.3 ± 0.6 ^a^	17.0 ± 1.0	35.7 ± 0.6	18.3 ± 1.2 ^e^
NL1	30.3 ± 0.6 ^b^	18.0 ± 0.0	36.3 ± 0.6	20.3 ± 0.6
NBL1	27.7 ± 1.2 ^b^	12.3 ± 0.6 ^c^	23.3 ± 0.6 ^d^	19.3 ± 1.0
NL5	35.3 ± 1.5	18.3 ± 0.6	40.0 ± 0.0	25.0 ± 1.0
NBL5	33.7 ± 0.6	13.0 ± 1.0	26.3 ± 1.5 ^d^	25.3 ± 1.2
NL7.5	35.7 ± 0.6	19.0 ± 1.0	37.7 ± 1.5	25.0 ± 1.0
NBL7.5	35.0 ± 1.0	14.3 ± 0.6	23.7 ± 2.5 ^d^	26.7 ± 0.6

The superscripts ^a–e^ in the columns represent a significant difference (*p* < 0.05) within the tested formulations.

**Table 4:** Composition of drug-free and lincomycin HCl-loaded borneol-ISG solutions.

Formulation Code	Lincomycin HCl (% *w*/*w*)	Borneol (% *w*/*w*)	NMP
NL1	1	-	99
NL5	5	-	95
NL7.5	7.5	-	92.5
NB40	-	40	60
NBL1	1	40	59
NBL5	5	40	55
NBL7.5	7.5	40	52.5

## Data Availability

The data presented in this study are available on request from the corresponding author.
